# Application of Multilayer Network Models in Bioinformatics

**DOI:** 10.3389/fgene.2021.664860

**Published:** 2021-03-31

**Authors:** Yuanyuan Lv, Shan Huang, Tianjiao Zhang, Bo Gao

**Affiliations:** ^1^Hainan Key Laboratory for Computational Science and Application, Hainan Normal University, Haikou, China; ^2^Yangtze Delta Region Institute, University of Electronic Science and Technology of China, Quzhou, China; ^3^Department of Neurology, The Second Affiliated Hospital of Harbin Medical University, Harbin, China; ^4^College of Information and Computer Engineering, Northeast Forestry University, Harbin, China; ^5^Department of Radiology, The Second Affiliated Hospital, Harbin Medical University, Harbin, China

**Keywords:** multilayer networks, bioinformatics, brain network structure, biological systems, chronological networks

## Abstract

Multilayer networks provide an efficient tool for studying complex systems, and with current, dramatic development of bioinformatics tools and accumulation of data, researchers have applied network concepts to all aspects of research problems in the field of biology. Addressing the combination of multilayer networks and bioinformatics, through summarizing the applications of multilayer network models in bioinformatics, this review classifies applications and presents a summary of the latest results. Among them, we classify the applications of multilayer networks according to the object of study. Furthermore, because of the systemic nature of biology, we classify the subjects into several hierarchical categories, such as cells, tissues, organs, and groups, according to the hierarchical nature of biological composition. On the basis of the complexity of biological systems, we selected brain research for a detailed explanation. We describe the application of multilayer networks and chronological networks in brain research to demonstrate the primary ideas associated with the application of multilayer networks in biological studies. Finally, we mention a quality assessment method focusing on multilayer and single-layer networks as an evaluation method emphasizing network studies.

## Introduction

In recent years, the formulation of multilayer networks has provided new methods for the study of multilevel network systems. Many biological systems comprise interconnected units that can be effectively modeled as networks, which are mathematical structures describing connections between points (Jing et al., 2019; Liu B. et al., 2020; Shao et al., 2020). Complex network systems provide powerful research tools and methods for studying biological fields (Kumari and Verma, 2020; Liu X. et al., 2020; Shao and Liu, 2020), from interactions between genes and proteins (Zhang et al., 2019; Li Z. et al., 2020; Zhai et al., 2020), to the study of tissue and organ functions (Yang et al., 2020), and even human brain study (Zhang J. et al., 2020). The complexity and evolutionary nature of biological systems enable the extensive application of multilayer networks and associated methods. Additionally, ecosystems and evolutionary systems evolve and change over time, and the corresponding network structures for these systems change correspondingly. Furthermore, the reasons for all these changes, particularly topological changes in the course of network structure change, and the importance of network feedback in network structure analyses are all topics worthy of exploration.

A network representation is a simplified description of a more complex, multifaceted system. A social system can include different types of interactions of different biological significance (e.g., cooperation or competition), while standard network approaches usually ignore these interactions or achieve integration through analyzing networks with different edge types separately. In bioinformatics studies using network structures, the progress of each biological system relies on the amount of data and/or new discoveries about unknown biological areas. For example, in the study of transcription-translation relationships between genes and proteins, genes and proteins are represented by nodes, and the correspondence between genes and proteins is represented by links in the network. Therefore, it is necessary to first understand the characteristics of each individual gene and protein, and the methods used to identify these relationships (Lin et al., 2019; Zhang D. et al., 2021; Zhang Z.Y. et al., 2021; Zulfiqar et al., 2021). Only then can the most relevant genes and corresponding proteins for a disease or symptom be identified through clustering or linkage analyses of the network, which further enables the investigation of target therapies for symptoms of disease (Zhu et al., 2018; Iliopoulos et al., 2020). These applications all rely on the data set and on the biological correspondence of genes and proteins.

The definition of a multilayer network varies slightly from one application to another. All edges and nodes in a single network are homogeneous, but in the real world, there is heterogeneity in both the objects represented by the nodes and the connections represented by the edges. Multilayer networks add additional tagging capabilities to traditional networks. That is, tagging terms are added to the traditional network, which can be understood as a composite of simple (single-layer) networks with different tags for complex networks. This is a relatively easy way to understand the definition of complex networks on different systems. Currently, according to different applications and subjects, multilayer networks can be divided into the following types:

(1) Multiplex networks: Networks in which the nodes on different layers are connected by inter-layer edges.
(a) In multi-relational networks, each layer represents a different type of interaction, i.e., different relationships are the distinguishing dimension for building a multilayer network, and the relationships are the tagged labels.
(b) In a temporal network, each layer encodes the same type of interaction at different time points or time windows. That is, time series (time windows) are the tagged labels between layers in a multilayer network.
(2) Interconnected networks: Nodes in different layers do not necessarily represent the same entities and inter-layer edges between different types of nodes may exist.
(a) The network of networks consists of subsystems, which are themselves networks. They are interconnected by interlayer edges between subsystem nodes.
(b) In a contextual network, each layer is interpreted to represent a different type of node. For example, interactions between males in one layer, interactions between females in another layer, and interactions between the sexes in a third layer. These interactions are represented by inter-layer edges.
(c) Spatial networks (also known as geometric networks) can be connected by ecological networks of the subjects moving between various locations.

Multilayer networks are currently used in various fields including physics, chemistry, biology, technology, finance, and social systems because of their inherent structural and functional characteristics. In this review, we briefly introduce the development of multilayer network concepts, techniques, and applications in bioinformatics by reviewing multilayer network applications in bioinformatics, and we summarize the outlook and development of multilayer networks in bioinformatics by analyzing current research.

## Multilayer Network Applications

**FIGURE 1:**
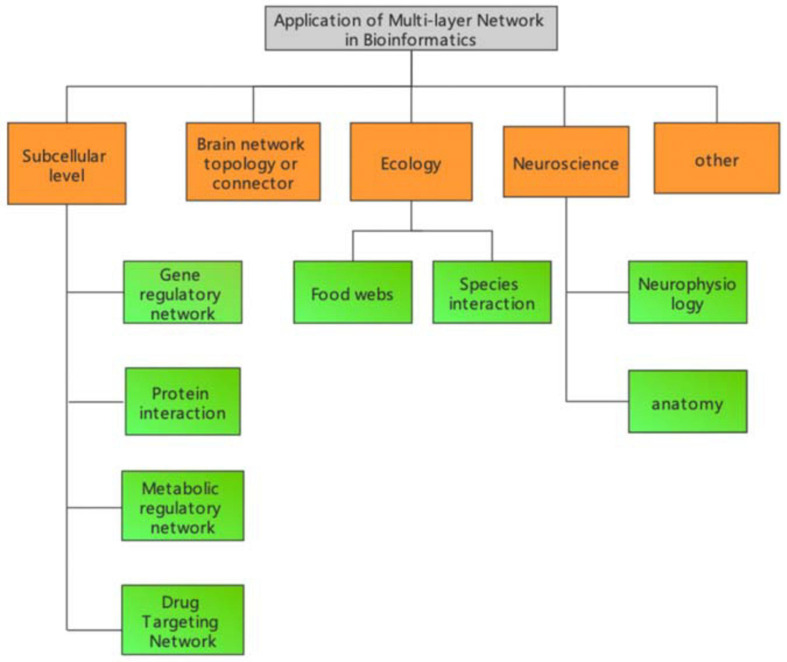
Multilayer network application area framework in bioinformatics.

The definition and methodology of multilayer network models in bioinformatics depends on the specific research problem. Organisms can be classified into different systems under different levels, and that system usually changes dynamically with time. Therefore, usually the representation of bioinformatics related networks varies depending on the specific biological system. In this review, we classify research topics into different categories according to the different levels of biological systems. As shown in Figure 1, multilayer networks in bioinformatics can be classified into five major categories.

As the understanding of DNA structure and function has gradually improved (Liu M.L. et al., 2020), understanding the relationships between genes and proteins, genes and disease, and disease and drugs has greatly evolved. For example, the correspondence between genes and disease has been mined through network structures, where the method utilizes a joint learning approach using the functional and connectivity patterns of proteins to predict disease-gene relationships using human interactome networks. In contrast to other data structures, interactomes are characterized by a high degree of incompleteness and lack of explicit negative knowledge, which makes prediction particularly challenging. To maximize potential information in the network, a second-order random walk procedure named random walker (RW^2) is applied in these studies. The random walker is able to learn rich representations of disease gene (or gene product) characteristics. This method has successfully compared with the best-known disease gene prediction systems and other state-of-the-art graph-based methods.

A large number of candidate disease-causing genes can be sequenced and checked for variation to help determine relationships between disease and genes (Zhang Z.M. et al., 2020). Many different computational methods have been developed to address this challenge. The observation that genes associated with similar diseases have a higher probability of interaction, many of these methods rely on the analysis of the topological properties of biological networks. However, the incomplete and noisy nature of biological networks is an important challenge. Two-step framework for disease gene ranking: (1) Construct a reliable functional connectivity network using sequence information and machine learning techniques. And (2) rank disease–gene relationships on the basis of that constructed functional connectivity network. Unlike other functional connectivity network-based frameworks that use functional connectivity networks based on the integration of various low-quality biological data, protein sequences can be used as comprehensive data to construct a reliable initial network. Additionally, the physicochemical properties of amino acids can be used to supplement hypotheses of protein function. In conclusion, our assessment of these methods indicates high efficiency and effectiveness for constructing functional linkage networks for disease genes (Wang et al., 2008; Jiang et al., 2010, 2013; Cheng et al., 2018; Zeng et al., 2018).

Gene function can also be determined by collecting biological data. For example, the *Drosophila* ovary epidermal cells (ECs) externally control the maintenance and progeny differentiation of germ line stem cells (GSC). In this study, the role of 173 EC genes that control GSC maintenance and progeny differentiation were identified using a *Drosophila in vivo* systemic RNAi approach (Zeng et al., 2016; Zou et al., 2016; Wang et al., 2019). Among the identified genes, 10 and 163 genes were required by ECs for GSC maintenance and progeny differentiation, respectively. The genes required for progeny differentiation were classified into different functional categories, including transcription, mRNA splicing, protein degradation, signal transduction, and cytoskeleton regulation (Cao et al., 2019). In addition, GSC progeny differentiation defects caused by defective ECs were often associated with BMP signaling elevation, indicating that preventing BMP signaling is a general functional feature of the differentiation niche. Finally, EC exon junction complex (EJC) components were identified as required for EC maintenance and the prevention of BMP signaling, and thus the promotion of GSC progeny differentiation. Therefore, this study identifies the major regulators of the differentiation niche and provides important insights into the external control of stem cell progeny differentiation.

Corresponding network structures for different biological data and specific subjects can also be designed to analyse specific biological systems (Zeng et al., 2016; Jiang et al., 2017; Liu et al., 2017). Currently, at the subcellular level, these networks mainly include gene regulatory networks (Wang et al., 2010; Ding et al., 2011; Jiang et al., 2014; Cheng et al., 2019; Konda et al., 2019; Liu L. et al., 2019; Mortezaeefar et al., 2019; Hong et al., 2020), protein functional networks (Guo et al., 2011, 2013, 2014; Sikandar et al., 2019; Tao et al., 2020; Liu et al., 2021), metabolic regulatory networks (Jin et al., 2020), and drug targeting networks (Wei et al., 2014; Ding et al., 2017, 2019, 2020a,b; Jin Q. et al., 2019; Jin S. et al., 2019; Srivastava et al., 2019; Zhao et al., 2019; Zeng et al., 2020).

The study of human brain functional and structural mechanisms using brain networks is also a hot field. Currently, research has mainly studied brain function by acquiring the brain waves of subjects, and the functional partitions of the brain have been predominantly obtained by functional experiments or magneto encephalography. This portion of our review will be introduced in detail in the next section.

Modern network theory is increasingly applied to neuroscience to understand neurophysiology and anatomy at different scales and under experimentally attainable physiological and pathophysiological conditions. The first attempt was made at the micro anatomical level of individual neurons. Watts and Strogatz analyzed the anatomical connections of the nervous system of *Caenorhabditis elegans* where neurons represent the nodes and synaptic or gap connections of a neural network. Their study revealed a highly clustered and efficient network, thus representing the first evidence of a real neural system with a small-world network. Later graph-theoretical approaches focused on morphological representations or dynamic correlations of the electrical stimulation activity of neuronal networks.

Network-based analyses have also been useful to address several questions in ecology and issues in conservation. The first study was carried out in a contextual network of so-called species interactions. Food webs are one of the fundamental issues in ecological studies, and despite the rather high variability detected in network structure, food webs present a complex topology similar to other types of real networks and host-parasite networks. One of the main advantages of these approaches is the direct assessment of the robustness and sensitivity of a given ecosystem to species loss or other perturbations. Another network type widely used in ecology is connected landscape mapping, where nodes typically represent patches on the landscape. The resulting spatial networks describe the linkages between processes and patterns that characterize the landscape, thus providing an effective way to assess important issues such as the effects of species dispersal or habitat loss.

Understanding the interactions between different species in a community and responses to environmental change is a central goal of ecology. However, defining the network structure of microbial communities is very challenging because of associated extremely diverse and unexplored states. Although recent developments in metagenomic technologies, such as high-throughput sequencing and functional gene arrays, have provided revolutionary tools for analyzing microbial community structure, it is still difficult to study network interactions in microbial communities based on high-throughput meta genomic data. A mathematical and bioinformatics framework for constructing molecular ecological networks (MENs) based on Random Matrix Theory (RMT) has been proposed. The remarkable feature of this approach compared with other network construction methods is that the network is automatically defined and robust to noise, thus providing a good solution to several common problems associated with high throughput.

## Applications of Multilayer Networks in Brain Research

The brain is the control center of most animal activities, and it has been the goal of many researchers to unravel the mystery of the brain and simulate the human brain with external devices such as computers. Before that, the structure and mechanism of the brain needs to be clarified, and it is costly to study the human brain because of its complexity. The human brain is a complex system organized by the structural and functional relationships among its components (Liu et al., 2018; Song et al., 2018; Liu G. et al., 2019). Recent experimental advances have led to unprecedented amounts of data that describe the structure and function of the brain, and it is now possible to model the brain as a network by measuring pairwise interactions between its various units. This modeling can be performed across multiple scales, where network nodes represent units of the brain, including proteins, neurons, brain regions, or other measurement units. Recording techniques such as functional magnetic resonance imaging (fMRI), magneto encephalography (MEG), and electroencephalography (EEG) are capable of capturing brain dynamics across time and across multiple frequency bands.

Recent neuroscience research has also exploited the versatility of multilayer frameworks to model complex relationships in neural data. For example, given fMRI and diffusion tensor imaging (DTI) for a single subject, a multilayer network can be constructed, with one layer representing the fMRI network and another layer representing the DTI network. Using the fMRI data, a functional network can be constructed in which the nodes represent brain regions and the edges represent the coherence between regional activities. On the basis of DTI data, a structural network can be constructed by dividing the brain into regions and then measuring the strength of physical connections between these regions. Finally, considering each network as a layer in a multilayer network, the edges of a brain region in the fMRI layer can be added to the DTI layer to form a multilayer network.

The brain is an inherently dynamic system, and the performance of cognition requires dynamic reconfiguration of a highly evolved network of brain regions, which interact in complex and transient communications. However, an accurate description of these reconfiguration processes during human cognitive function remains elusive (Liu and Jiang, 2016). Therefore, many studies have used temporal networks to investigate the dynamic cluster structure of brain networks and reveal the underlying human brain dynamic changes during learning. Temporal networks that contain temporal information have the advantage of retaining the full information of the data without aggregating connections into individual networks.

When we complete different cognitive vision tasks, we subdivide the regional time series into time windows of variable size, and determine the impact of the time window size on the observed dynamics. Specifically, we applied a multilayer community detection algorithm to identify temporal communities, and we computed network flexibility to quantify the changes in these communities over time. Within our frequency band of interest, large and small time windows were associated with the brain network flexibility within a narrow range, while medium time windows were associated with broad network flexibility. Using medium time windows of 75–100 s, we identified brain regions with low flexibility (considered core regions and observed in visual and attentional areas) and brain regions with high flexibility (considered peripheral regions and observed in subcortical and temporal lobe regions) by comparison with appropriate control dynamic network models. In general, this work demonstrates the effect of time window size on the network dynamics observed during task execution, providing practical considerations when selecting time windows in dynamic network analysis. More generally, this work reveals organizing principles for functional brain connections that are inaccessible to static network approaches.

The hypothesis that human executive functions arise from the dynamic interactions of multiple networks has been tested in previous research (Ding et al., 2019). To corroborate this research, we investigated a key executive function (FCD), namely arbitrary visuomotor mapping. MEG and intracranial EEG were recorded using high gamma activity brain connectivity analysis. We then generated visuomotor mapping using the dynamic interactions of three partially overlapping cortico-cortical and cortico-subcortical functional connectivity (FC) networks. First, visual and parietal regions were coordinated with sensorimotor and premotor regions. Second, dorsal fronto parietal circuits dominated by sensorimotor and associative frontal striatal networks were incorporated. Finally, bilateral sensory-motor areas were coordinated with the cortico-cortical hemisphere between the left fronto parietal network and the visual areas. Our study argued that these networks reflect the processing of visual information, the emergence of visuomotor plans, and the processing of somatosensory responses or action outcomes. Thus, our study demonstrates that visuomotor integration exists in the dynamic reconfiguration of multiple cortico-cortical and cortico-subcortical FC networks. More generally, the approach demonstrates that optokinetic-related FC is unstable and shows task performance-related switching dynamics and regional flexibility on a time scale. In addition, our optokinetic-related FC has sparse connectivity with a density of 10%. Taken together, these findings shed light on the relationship between dynamic network reconfiguration and short-time executive function and provide a candidate start point for the better understanding of cognitive structure.

A vast number of multilayer network applications exists in bioinformatics, but the application of multilayer networks in any subfield of bioinformatics still relies on the acquisition and accumulation of bioinformatics data, and brain research is no exception. Therefore, interdisciplinary collaboration is a very efficient and necessary option. Brain structure and functionality are gradually understood, driven by brain data acquisition. According to these studies, the dynamic modeling of brain function by combining temporal dimensions is an effective means of study. Perhaps as research progresses, new data dimensions will be added (Wang et al., 2018, 2020; Wei et al., 2018a; Ding et al., 2019; Liu B. et al., 2019; Su et al., 2019b; Dao et al., 2020; Li J. et al., 2020; Lv et al., 2020).

## Conclusion and Perspectives

Multilayer (complex) networks have been an effective tool for studying complex problems in recent years and are currently being used in a variety of fields. As systems biology develops, multilayer networks are applicable to many aspects and research areas within the field. Because of dataset availability, these networks are currently more often applied to genetics and brain research. However, as research progresses, it should become easier to unravel structural and functional fogs in biology on one hand, and on the other hand, research in this area will prove beneficial to the understanding of biological principles in general to better serve all people. In view of current research status, our review has presented the following ideas and prospects:

(1) The development of biology is promoted by the joint development of various fields, and the application of multilayer networks in bioinformatics depends on the accumulation of biological data and the development of computer-related theories. Therefore, as an interdisciplinary subject, it needs the collaborative work of interdisciplinary experts.
(2) Because of the complexity and dynamic change of biological systems, time-series multilayer networks with the addition of temporal information will have more and more applications in the simulation of dynamic processes in the study of genes, disease, drug discovery, and brain research.
(3) Exploring the communication mode between tissue cells in the form of multi-layer network is to study the interaction (functionality) between structures on the basis of the network represented by the structure.

In addition to the structural and functional aspects of multilayer network research, methods to efficiently evaluate and assess the results of multilayer networks remains an importan tissue. The evaluation of the algorithmic complexity of multilayer networks has been proposed to assess if and when the multilayer representation of a system is qualitatively superior to classical single-layer aggregation network approaches (Wei et al., 2017a,b,c, 2018b, 2019; Su et al., 2019a, 2020a,b; Wang D. et al., 2021; Wang H. et al., 2021; Zhao et al., 2017).

## Author Contributions

YL contributed to conception and design of the study and wrote the first draft of the manuscript. SH organized the database. BG performed the statistical analysis. TZ, YL, and BG wrote sections of the manuscript. All authors contributed to manuscript revision, read, and approved the submitted version.

## Conflict of Interest

The authors declare that the research was conducted in the absence of any commercial or financial relationships that could be construed as a potential conflict of interest.
